# Murine Typhus and Leptospirosis as Causes of Acute Undifferentiated Fever, Indonesia

**DOI:** 10.3201/eid1506.081405

**Published:** 2009-06

**Authors:** M. Hussein Gasem, Jiri F.P. Wagenaar, Marga G.A. Goris, Mateus S. Adi, Bambang B. Isbandrio, Rudy A. Hartskeerl, Jean-Marc Rolain, Didier Raoult, Eric C.M. van Gorp

**Affiliations:** Diponegoro University, Semarang, Indonesia (M.H. Gasem, M.S. Adi, B.B. Isbandrio); Slotervaart Hospital, Amsterdam, the Netherlands (J.F.P. Wagenaar, E.C.M. van Gorp); Royal Tropical Institute, Amsterdam (M.G.A. Goris, R.A. Hartskeeri); Université de la Méditerranée, Marseille, France (J.M. Rolain, D. Raoult); 1 These authors equally contributed to this article.

**Keywords:** Fever of unknown origin, Rickettsia, vector-borne infections, Rickettsia typhi, murine typhus, leptospirosis, leptospira, Java, Indonesia, dispatch

## Abstract

To investigate rickettsioses and leptospirosis among urban residents of Semarang, Indonesia, we tested the blood of 137 patients with fever. Evidence of *Rickettsia typhi,* the agent of murine typhus, was found in 9 patients. Another 9 patients showed inconclusive serologic results. Thirteen patients received a diagnosis of leptospirosis. No dual infections were detected.

Fever is one of the main reasons for seeking medical attention in Indonesia. The cause of fever usually remains obscure because of limited laboratory diagnostic facilities and expertise in performing laboratory confirmation. Favorable environmental conditions mean that both rickettsiosis and leptospirosis are considered endemic in Indonesia and may result in clinically indistinguishable cases of acute undifferentiated fever (AUF). Serosurveys conducted on Java, Sumatra, and islands in eastern Indonesia identified antibodies to *Rickettsia typhi* (murine typhus), to *Orientia tsutsugamushi* (scrub typhus), and to members of the spotted fever group rickettsia (SFGR) in healthy persons (*1*–*3*). In addition, several investigations reported leptospirosis as cause of AUF in Indonesia (*4*,*5*).

Murine typhus and leptospirosis are likely to share routes of transmission in an urban setting where rats are abundant. The main vector for *R. typhi* is the Asiatic rat flea (*Xenopsylla cheopsis*). Humans usually become infected when *R. typhi*–infected flea feces contaminates excoriated skin or is inhaled. Leptospirosis is mainly spread by rats and other small mammals, which shed the bacteria through their urine into the environment. Humans are infected through mucous membranes, conjunctivae, or abraded skin.

The clinical features of mild leptospirosis and murine typhus are nonspecific. Generally, patients with murine typhus exhibit fever, headache, and a rash, although the latter is often absent. Renal failure, jaundice, and hemorrhages are the classic symptoms of severe leptospirosis; fever, headache, and myalgia may be the only exhibited symptoms of mild disease. Dual infections with murine typhus are reported to occur in Southeast Asia and may complicate treatment and clinical course (*6*,*7*). In this study, we attempted to find evidence for acute rickettsial disease, leptospirosis, and dual infections among patients with AUF in Indonesia, where risk factors for both diseases are present.

## The Study

The study was based in Semarang, a large harbor city in central Java. Consecutive outpatients were recruited at 2 primary healthcare centers and hospitalized patients at a governmental referral center (Dr. Kariadi University Hospital, Department of Internal Medicine). All eligible AUF patients (>5 years of age) were included who met the following criteria: fever >38°C (central) for <14 days with no apparent other disease. After informed consent was obtained, a blood sample was taken. A convalescent-phase sample was drawn after ≈14 days. The study was approved by the local medical ethical committee.

A specific microimmunofluorescent antibody (IFA) assay for *Rickettsia* spp. was performed in Marseille, France, by using whole-cell antigens of *O. tsutsugamushi*, *R. japonica*, *R. heilongjiangensis*, *R. slovaca*, *R. honei*, *R. conorii* subsp. *indica*, *Rickettsia* ATI, *R. helvetica*, *R. felis*, *R. typhi*, and *R. prowazekii* . The assay results were considered positive when 1) antibody titers were >256 for immunoglobulin (Ig) G and >64 for IgM, or 2) seroconversion was observed, or 3) a >4-fold increase in titers between the acute-phase and the convalescent-phase serum specimen was detected. Serologic analysis for leptospirosis was performed in Semarang, Indonesia. Crosschecks and PCR were performed in Amsterdam, the Netherlands. Convalescent-phase samples were screened with the LeptoTek Dri Dot (bioMérieux, Marcy l’Etoile, France). All positive samples were tested by the microscopic agglutination test (MAT) and IgG ELISA (*8*).

Additionally, a real-time PCR specifically targeting the *secY* gene of pathogenic *Leptospira* spp. (*9*) was performed on all samples. For the MAT, a panel of 31 serovars was used containing 28 pathogenic and 3 nonpathogenic serovars. For patient samples tested by ELISA or MAT, a titer >320 on a single sample was considered positive; also considered positive were those samples that showed seroconversion or a >4-fold increase in titers between paired samples, as well as any patient sample with a positive PCR, irrespective of the serologic result. All samples were run in parallel.

From February 2005 through February 2006, 137 AUF patients were included: 67 hospitalized patients and 70 outpatients. A convalescent-phase sample was available for 106 (77%) patients. The main symptoms were headache (85%), myalgia (70%), nausea (64%), cough (44%), and abdominal pain (38%).

Murine typhus and leptospirosis were found to cause AUF in this clinical series (Appendix Table). In total, 9 patients (7%) had evidence of an acute infection with *R. typhi*; none showed a rash. Murine typhus could be diagnosed in 6 (9%) of 67 hospitalized patients; 3 (4%) of 70 outpatients had acute murine typhus. Another 9 (7%) patients showed inconclusive *R. typhi* serologic results. One patient showed evidence of a past infection with *R. typhi* (IFA IgG/IgM titer 128/0 in both serum specimens). Evidence for acute infection with *O. tsutsugamushi* or SFGR was not found.

Leptospirosis was diagnosed in 13 (10%) of 137 patients; results for 2 of these patients were positive only by PCR. Eleven leptospirosis patients were recruited in the hospital; 2 patients were recruited outside the hospital. Consequently, the percentage of AUF caused by leptospirosis in hospitalized patients was 16% and in outpatients 3%. The most frequently identified serogroup by MAT was Bataviae (5 cases). No dual infections were detected; however, 3 (23%) leptospirosis patients showed titers in the *R. typhi* IFA assay.

## Conclusions

We report that murine typhus and leptospirosis are important causes of AUF in Semarang, Indonesia. A previous study from rural Thailand identified both diseases in 2.8% and 36.9% of AUF cases, respectively (*6*,*7*). In Vientiane, the capital of Laos, *R. typhi* was reported to cause fever in 9.6% of investigated persons, results that closely resembling our data (*10*). Unfortunately, leptospirosis was not investigated.

In the present study, we expected leptospirosis to be a cause of AUF because the Dr. Kariadi University Hospital admits ≈50 severe cases each year. These cases were not included in the study because of the high clinical suspicion of leptospirosis on admission with jaundice, azotemia, and/or bleeding. A definite diagnosis of murine typhus and leptospirosis co-infections could not be made, but in 3 cases this scenario was plausible. We did not find evidence for scrub typhus, which we expected, because *O. tsutsugamushi* transmission occurs primarily in rural areas (*11*). Although SFGR have been reported in Southeast Asia and proof for their presence in Indonesia is accumulating (*2*,*12*), these rickettsia were not identified as a cause of AUF in the present study.

From an epidemiologic point of view, Semarang, Indonesia, seems to encompass environmental circumstances that are prerequisites for *R. typhi* and leptospirosis transmission. Previous studies have shown that murine typhus is particularly prevalent in tropical port cities where rats are abundant (*13*,*14*). In the Indonesian urban situation, *R. rattus* and *R. norvegicus* rats are likely to be the main hosts harboring *R. typhi*–infected *X. cheopsis* fleas (*12*,*15*). These rats are also likely to be the maintenance hosts for pathogenic *Leptospira* spp. in Indonesia. In fact, the identified serogroups are commonly associated with rats.

Although serologic analysis might be hampered by cross-reactions and test sensitivity issues, we believe that our data are representative for the area. The chosen cut-off values are unlikely to cause false-positive results in a disease-endemic setting. In regard to leptospirosis serologic analysis, we used a wide panel for the MAT. This panel included serovars recommended by the World Health Organization and serovars that were previously isolated in Indonesia. Moreover, most serogroups were represented in our panel, and cross-reactions are likely to detect missing serovars.

Because of nonspecific clinical features, both diseases are difficult to diagnose on clinical grounds only. Misdiagnosis can lead to aberrant use of antimicrobial drugs and other pharmaceuticals. Therefore, rapid, cheap, and reliable diagnostic tests are needed to support clinical decision making.

## Supplementary Material

Appendix Table Clinical and laboratory data of patients with rickettsioses and leptospirosis, Indonesia, February 2005-February 2006*
