# Supplementary material for: Murine Typhus and Leptospirosis as Causes of Acute Undifferentiated Fever, Indonesia
Source: Emerg Infect Dis. 2009 Jun;15(6):975–7. doi: 10.3201/eid1506.081405 (PMC2727336; doi:10.3201/eid1506.081405)
Supplement: Appendix Table — Clinical and laboratory data of patients with rickettsioses and leptospirosis, Indonesia, February 2005-February 2006* [file 08-1405_appT-s1.pdf]

Publisher: CDC; Journal: Emerging Infectious Diseases
Article Type: Dispatch; Volume: 15; Issue: 6; Year: 2009; Article ID: 08-1405
DOI: 10.3201/eid1506.081405; TOC Head: Dispatch

Appendix Table. Clinical and laboratory data of patients with rickettsioses and leptospirosis, Indonesia, February 2005–February 2006*

Disease/ patient no.	Age, y/sex	Month of illness onset	*Rickettsia typhi* IFA (IgG/IgM)		MAT		Leptospirosis PCR result	Clinical features and treatment
			Admission sample	Convalescent-phase sample	Highest titer pathogenic serovar	Putative infecting serogroup		
Murine typhus								
7	49/M	Feb	0/0	1,024/1,024	NA	NA	–	4-d fever, myalgia, headache, chills, cough, abdominal pain; Rx: ciprofloxacin
10	45/M	Mar	512/1,024	512/1,024	NA	NA	–	7-d fever, myalgia, headache, nausea, abdominal pain; Rx: ciprofloxacin
12	46/F	Mar	2,048/512	2,048/512	NA	NA	–	3-d fever, headache, chills, nausea, vomiting, hepatomegaly; Rx: ciprofloxacin
18	15/M	Apr	256/64	256/64	NA	NA	–	3-d fever, headache, sore throat, abdominal pain; Rx: cefadroxyl
24	46/F	Apr	128/512	1,024/1,024	NA	NA	–	7-d fever, headache, nausea, abdominal pain; Rx: ciprofloxacin
63	68/F	Nov	2,048/256	2,048/512	NA	NA	–	3-d fever, myalgia, headache, chills, nausea, dyspnea; Rx: ciprofloxacin
1107	13/F	Feb	0/32	1,024/512	NA	NA	–	2-d fever, headache, anorexia; Rx: cotrimoxazol
1112	25/M	Feb	256/64	NA	NA	NA	–	2-d fever, myalgia, headache, anorexia, sore throat, cough, abdominal pain, calf pain; Rx: amoxicillin
1123	8/F	Mar	0/64	512/0	NA	NA	–	1-d fever, headache, sore throat, nausea, vomiting, abdominal pain; Rx: amoxicillin
Possible murine typhus								
2	17/F	Feb	0/0	0/64	NA	NA	–	5-d fever, myalgia, headache, chills, cough sore throat; no antimicrobial drug
5	31/F	Feb	0/0	128/0	NA	NA	–	5-d fever, myalgia, headache, chills, nausea; no antimicrobial drug
53	22/F	Aug	128/32	128/64	NA	NA	–	4-d fever, myalgia, headache, nausea, vomiting, abdominal pain, diarrhea; no antimicrobial drug
62	17/M	Nov	128/64	128/64	NA	NA	–	4-d fever, myalgia, headache, chills, sore throat, nausea, vomiting, abdominal pain, petechiae; no antimicrobial drug
69	46/M	Dec	0/128	0/128	NA	NA	–	9-d fever, myalgia, headache, chills, nausea, abdominal pain; Rx: ciprofloxacin

1104	14/M	Feb	128/64	128/64	NA	NA	–	1-d fever, headache; Rx: amoxicillin
1105	11/F	Feb	128/32	NA	NA	NA	–	2-d fever, myalgia, headache, nausea, vomiting, abdominal pain; Rx: amoxicillin
1113	70/F	Feb	0/0	0/64	NA	NA	–	1-d fever, myalgia, headache, sore throat, cough, calve pain; Rx: amoxicillin
1132	7/M	Mar	256/0	NA	NA	NA	NA‡	3-d fever, headache, sore throat, nausea, vomiting, conjunctival suffusion, petechiae; Rx: amoxicillin
Leptospirosis								
4	27/M	Feb	–	–	80†	Javanica	–	2-d fever, myalgia, headache, chills, nausea, vomiting, abdominal pain, calve pain, hepatomegaly; Rx: cefotaxime
14	63/M	Mar	128/0	128/0	1,280	Hebdomadis	–	3-d fever, myalgia, headache, nausea, vomiting, abdominal pain; Rx: ciprofloxacin
25	41/F	May	–	–	80†	Bataviae	–	2-d fever, myalgia, headache, chills, nausea, vomiting; no antimicrobial drug
29	49/M	May	–	–	160†	Bataviae	–	4-d fever, myalgia, headache, chills, diarrhea, conjunctival suffusion; Rx: ciprofloxacin and PP
32	40/M	May	–	–	320	Bataviae	–	5-d fever, myalgia, headache, nausea, vomiting, abdominal pain, petechiae; no antimicrobial drug
37	50/M	May	–	–	40†	NC	–	5-d fever, headache, chills, cough, nausea, vomiting, abdominal pain, diarrhea; Rx: PP
40	63/M	Jun	–	–	–	NC	+	2 d fever, myalgia, headache, nausea, vomiting, abdominal pain, calve pain; Rx: PP
48	16/M	Jun	–	–	640	Saxkoebing	–	2-d fever, myalgia, headache, chills, petechiae; no antimicrobial drug
1152	17/M	Mar	–	–	160†	Bataviae	–	2-d fever, myalgia, headache, chills, sore throat, cough, nausea; Rx: amoxicillin, tetracycline, cotrimoxazol
1182	58/F	Jan	–	–	320§	NC	+	2-d fever, myalgia, headache, cough; Rx: cotrimoxazol
Possible co-infection								
33	38/M	May	1,024/0	1,024/0	320	Icterohemorrhagiae	–	5-d fever, myalgia, headache, nausea, abdominal pain, diarrhea, calve pain; Rx: cefotaxime

43	39/F	Jul	0/0	0/256	640	Bataviae	+	5-d fever, myalgia, headache, chills, nausea, vomiting, jaundice, calve pain; Rx: PP
60	37/M	Oct	0/64	64/64	–	NC	+	5-d fever, myalgia, headache, nausea, abdominal pain; no antimicrobial drug

*IFA, microimmunofluorescent antibody; Ig, immunoglobulin; MAT, microscopic agglutination test; NA, not available; NC, not classifiable; PP, procaine penicillin; Rx, medical prescription.
†Patient seroconverted from negative (<20) to a positive titer by MAT or ELISA.
‡Insufficient sample to perform PCR; leptospirosis highly suspected in patient.
§IgG ELISA result; the highest titer by MAT was <40.
